# Notes on Medical Matters and Medical Men in Paris

**Published:** 1847-12

**Authors:** David W. Yandell

**Affiliations:** Louisville, Ky.; Paris


### WESTERN JOURNAL

OF

### MEDICINE AND SURGERY.

DECEMBER, 1847.

### Art. I.—*Notes on Medical Matters and Medical Men in Paris.* By David W. Yandell, M.D., of Louisville, Ky.

### LECTURE ON THE DISEASES OF THE GENITAL ORGANS OF THE FEMALE.

Delivered at Lourcine Hospital, by M. Hugier.

*Catarrh of the Uterus.*—The disease of which we propose to speak this morning has been the object of our special attention. After long researches, we think we have arrived at results calculated to clear up certain hitherto obscure points relative to the history of catarrh of the uterus. If we consult the authors who have written upon the subject which occupies us, we find them vague, and not unfrequently guilty of errors which are not in all instances without importance. For example, catarrh of the uterus is generally regarded as the consequence of an acute or chronic inflammation—an affection of the internal membrane of this organ—and to this affection various species of discharges are often attributed, when in reality they have an entirely different origin. In our opinion, the word catarrh is not applicable to a disease of the lining membrane of the body of the uterus, but is clearly and distinctly an affection seated in certain points of the neck, or, in other words, in the follicles of this organ. We consider the discharge proper to catarrh of the uterus a peculiar liquid, very distinct from other kinds of discharges. This liquid is thready, thick, viscid, consistent, of an albuminous or vitreous aspect, and endowed with alkaline properties.

Before entering directly into our subject, allow us to offer you some considerations upon the various discharges which proceed from the genital organs of the female. These preliminaries will be of service to you; they will aid you in understanding the history of uterine catarrh, and will especially render more easy of appreciation the characters which are proper to discharges symptomatic of this affection; for there are many discharges which have been frequently regarded as belonging to catarrh which are entirely foreign to it, and which are vaguely designated under the names of fluor albus, leucorrhea, blennorrhagia, etc. This want of exactness, this confusion in language, is not without inconvenience; it denotes a defect of observation injurious in its therapeutical consequences. In order properly to appreciate the value of discharges in general, we will examine them one by one as regards their seat, and to do so we will divide them as follows:

1. *Vulvar discharges.*—These proceed from the mucous membrane of the major and minor labia, clitoris, and constrictor vaginæ. The liquid which constitutes them may be more or less abundant, thick or aqueous, white or greenish white, mucous or purulent; it is ordinarily acid. This kind of discharge arises either from a local inflammatory or atonic state, simple or blennorrhagic.

2. To these discharges we will add those which are the product of the secretion of the vulvo-vaginal glands, and which may be more or less abundant, simply mucous or purulent; but in these various cases the liquids never offer the properties which characterize the discharges of uterine catarrh.

3. *Urethral discharges.*—A simple or specific urethritis may furnish, by the orifice of the urethra, a more or less copious, thick, mucous, purulent, or muco-purulent discharge, but never similar to the liquid secreted by the follicular apparatus of the cervix uteri.

4. *Vaginal discharges.*—The same remark is applicable to the fluids secreted by the mucous membrane of the vagina. In fact, the vaginal discharges—the nature of which varies in other respects, and which recognise as their cause an inflammatory or virulent affection, or even an atonic state of the vagina, as that which obtains in veritable leucorrhea—these discharges, we say, never present that transparent albuminous aspect, that viscid, thready, elastic character, and, finally, that alkaline property, which we constantly find in catarrh of the uterus. It may indeed happen that these vaginal discharges may offer characters similar to those possessed by the liquids of catarrh; but in this case it arises from a mixture of two liquids, the one coming from the mucous membrane of the vagina, the other from the seat of the affection which occupies us, that is to say, from the follicles of the cervix; so that if it were possible to separate the different products, we should find the characters proper to each; consequently it follows that this is but a coincidence or complication of two affections, which are not, however, the less distinct the one from the other.

5. *Uterine discharges.*—We are now arrived at the essential point of the question. It does not suffice to have studied the discharges which do not arise from the uterus in order to believe that we now only have to do with those that are proper to uterine catarrh; for this is knowledge which does not furnish all the elements necessary to our diagnosis. We must push our investigations still farther, and in doing this we will establish an important distinction between that which concerns the uterus, and the products of secretion.

We must consider the uterus as divided into two parts, the neck and body. This distinction, besides being true as regards its configuration taken as a whole, is not the less true as regards its anatomical structure; with this difference, as we will see further on, the existence of distinct physiological and pathological phenomena of great importance. In fact, the body and neck of the uterus are distinct in their functions and diseases, the liquids secreted by the neck differing from those which are produced by the internal membrane of the body. It is not this which offers the anatomical seat of the discharge which constitutes uterine catarrh, while it is the neck which is the secreting organ of this fluid.

Let us now carefully study the anatomical and physiological differences which distinguish the two constituent parts of the organ of gestation, in order more satisfactorily to appreciate the pathological phenomena.

The surface of the neck offers numerous folds, which are intended to facilitate its dilatation during labor; the internal surface of the body is, on the contrary, smooth, polished, and in nowise wrinkled. When this organ, as obtains in pregnancy, augments in volume, its expansion is neither instantaneous nor on the other hand passive; it is effected in consequence of a movement of general nutrition, gradually and insensibly.

If we examine the interior of the cavity of the neck, we ordinarily find a variable quantity of mucous matter, transparent, viscid, and thready between the fingers. The cavity of the body offers nothing similar; the liquid which it may contain is more or less thick and serous, never resembling that of the neck. It is in the cavity of this organ, and upon certain points of the surface of the vagina, that we often perceive a number of mucous follicles, called *glandulæ nabothi*, shut follicles, charged with secreting the thick, thready, gelatinous mucus of which we have just spoken. But these little glandular bodies never show themselves in the cavity of the body; and even if placed there, what end could they serve? Would not their presence be useless, or even hurtful? Would not the follicular liquid secreted by them in the cavity of the body often offer an obstacle to fecundation? and would it not favor the escape of the ovum, and render its exit easy? Placed, on the contrary, in the cavity of the neck, secreted, perhaps, more abundantly after the act of conception, the follicular mucus obstructing and obliterating the cavity of the neck, facilitates the engrafting of the ovum, which is thus prevented from passing out. The obliteration in the mode of which we are speaking may take place to such an extent as to prove, in certain cases, a true cause of sterility, by opposing itself to the penetration of the spermatic fluid during the act of copulation.

The follicular liquid shows itself useful again during labor. In moistening the cavity of the neck, it favors its dilatation, facilitates the passage of the head of the child, and consequently renders the woman less liable to those more or less serious lacerations to which she is sometimes exposed. But whatever may be the physiological value of the liquid and of the follicular organ, it is certain that the secretory apparatus of which we have spoken exists only in the neck; in our numerous researches we have never been able to detect its presence in the cavity of the uterus. Without doubt, products of secretion do exist in this cavity, but these products, whatever they may be, always differ from the liquids furnished by the follicles of the neck. One does not see, we repeat it, upon the internal membrane of the body of the uterus anything similar to the mucous follicles presented by the neck. The small glandlike bodies that are found there are nothing more than ramifying submucous canals, and the glands pointed out by Weber are themselves but the products of the membrana decidua, and consequently do not properly belong to the uterus. In fact, what *is* the internal membrane of this organ? What is the general opinion of anatomists concerning it? Now, it is evident that if the greater part of them contest the existence of a mucous membrane lining the cavity of the uterus, they do not hesitate to admit it for the cavity of the neck; such is the opinion of Heister, Méry, Azaguidi, Morgagni, Ribes, Chaussier, Dugès, and Madame Boivin, whose works and manner of viewing the question are of great value.

These authors assimilate the uterine membrane to the serous membrane of the heart, and do not recognise a mucous membrane except upon the point of insertion of the placenta. P. Dubois and Cruveilhier, it is true, believe in the existence of a true mucous uterine membrane; but we must observe that these authors base their opinion only upon analogies, and in no instance upon anatomical demonstration. For ourselves, we will say, relative to the question, that the cavity of the body of the uterus is lined by a mucous membrane, but that this membrane does not exist except in a rudimentary state, while the internal membrane of the neck offers all the elements which characterize a complete mucous membrane.

The same anatomists that we have mentioned, Nabothi himself, and, very recently, Martin-Saint-Ange and Grimaud, describing the follicles in question, recognise their existence only in the neck, never having seen them elsewhere. Certain special observations come still to the support of this view. It is thus that Morgagni, Dugès and Madame Boivin and many other anatomists, having had occasion to examine the uterus in women who died during the period of menstruation, have signalized as very sensible, and more or less voluminous, the follicular bodies of which we speak; but they have remarked their existence only in the neck, never in the body.

These, now, are circumstances which would have revealed similar follicles in the cavity of the body if they had really existed there. Moreover, the liquid secreted by the internal membrane of the uterus, which constitutes what is sometimes called the false waters, resembles in appearance peritoneal serum, but is never thick, thready, albuminous—in a word, similar to the mucus secreted by the follicles. The same remark is applicable to the nature of the purely aqueous liquids that we see discharged in an intermittent manner in certain cases of insertion of the placenta into the neck. If we examine, moreover, that which occurs in obliteration of the cavity of the uterine neck, the observation again confirms our opinion. In effect, when it is the inferior orifice which is obliterated, we find in the uterine cavity an aqueous liquid mixed with another fluid which is mucous, thready, transparent. On the contrary, in cases of obliteration of the superior orifice, no similar mixture is found; the liquid which exists then in the cavity of the body preserves its ordinary characters; it is aqueous, serous, never viscid, thready or albuminous, and in no respect resembles the fluid contained in the neck. We will add, that whatever may be the lesions of the uterus, the cavity of this organ has never shown us liquids analogous to those which result from the secretion of the follicles, and of the characters of which you may gain an extremely good idea from those patients laboring under catarrh of the uterus, which we shall show you after our lecture.

To terminate our rapid glance at the various secretions furnished by the genital organs of the female, which you must not confound with the discharge proper to the disease which occupies us, we should mention the secretion from the internal membrane of the uterus—discharges symptomatic of various affections of this organ; we should indicate, finally, the liquids secreted by the appendages of the uterus, the Fallopian tubes, attendant upon the numerous affections of which these organs are susceptible, hypersecretion, dropsies, cysts, etc. But in these various cases the discharges differ essentially from the products of the secretion of the follicles of the neck, and cannot be confounded with them.

To recapitulate: the genital organs of the female give place to discharges which, viewed as regards their seat, may be divided as follows: 1st, vulvar discharges; 2d, discharges coming from the vulvo-vaginal secretory apparatus; 3d, urethral; 4th, vaginal; 5th, uterine, which may themselves be subdivided into those which proceed from the neck, and those which are furnished by the body; and, 6th, discharges originating in the appendages to the uterus. Now, of all these various discharges, those only belong to the affection designated *catarrh of the uterus* which offer the characters that we have indicated, and which are seated solely in the follicular apparatus of the neck. It is then the neck—its follicles—that we should regard as the true seat of the disease which now engages us.

The different considerations which we have presented will assist you in understanding certain points relative to the history of the disease, so that we may now say something of its etiology. Catarrh of the uterus is observed principally in young women who abuse sexual pleasures, in those of a lymphatic temperament, with soft red hair, or in those who have no other mark of the *brune* than the color of their hair. Cold and moist climates also predispose to this affection. Masturbation, excessive coition and pregnancy are not without influence in the production of uterine catarrh; but it sometimes happens, when it depends upon pregnancy, that it disappears after delivery, and is consequently but a transient affection. There are besides many diseases that should be regarded as predisposing causes, and especially slow, chronic inflammation of the uterus, which determines in this organ irritative fluxionary movements, and consequently a hypersecretion of the follicular apparatus.

Certain vices of the humors—a scrofulous affection, for example—being capable of deteriorating the female economy—of developing in it a sort of mucous constitution—may create and sustain a catarrh to which the name of scrofulous catarrh might be given. We will make the same remark of blennorrhagic and syphilitic diseases, the morbid product of which may have attained the follicles of the neck, and there given rise to a virulent catarrhal hypersecretion. It sometimes occurs, in cases of this nature, that the specific cause being removed, the catarrhal affection dependent upon it also disappears, though this, on the other hand, may continue after the removal of the cause. The etiological causes which we have just enumerated being established, we will return again to the product of the secretion of the follicular apparatus—a product whose importance we should bear in mind, as being the essential element of the disease.

The follicles which furnish the liquid proper to catarrh of the uterus are seated, as we have already remarked, at the neck. In the cavity of this part of the organ they are less numerous towards its orifice than upon its middle portion; they are also found disseminated on the extra-uterine surface of the cervix, and these consequently being, like the first, susceptible of disease, may in this case give rise to a form of catarrh which might very properly be designated by the term of extra-uterine catarrh. Of these follicles, some are superficial, that is to say, placed under the mucous membrane, while others are deep, situated in the substance even of the walls of the neck, at a line and even two lines in depth in the proper tissue of the uterus. The glandular corpuscles of which we have spoken, irritated and inflamed, have often been described under the vague name of granular metritis, without the writers knowing to what anatomical lesion it was to be attributed.

Let us now establish an important distinction between the normal or physiological, and the pathological products of these follicles. We will first remark, that there exists in them normally a certain quantity of mucus, secreted by the follicles in the cavity of the neck; but in the physiological condition of the organ, the mucus is always transparent, resembling very much a vitreous body. When this liquid is secreted a little more abundantly than ordinary, it may escape by the vagina, and cause certain women to think that they are laboring under fluor albus. In cases of this sort there exists but a simple hypersecretion of the follicles, that is, a state bordering upon catarrh properly so called. It manifests itself without redness, inflammation or tumefaction of the neck of the uterus; it is met with principally in women who have had several children, or in those whose uterus has become the seat of an active hyperæmia. To establish, then, the existence of a catarrh, it is not only necessary that there be an augmentation in the liquid secreted, but also that it present either an opaque, a dull white, or a purulent aspect. This is its differential character.

Let us now examine this morbid product as regards its contagious properties:

1. *Physical Characters.*—The liquid may be observed at the vulva, in the vagina, and at the neck. When examined at the vulva, we see at its orifice a flake of thick, elastic, thready matter, which is detached with difficulty, unctuous to the touch, and therefore slightly sensible to the finger. In some cases the neck seems swollen, dilated like a bubble. To the touch the neck appears then supple and pulpy, as if it were softened; and if you attempt by the aid of the forceps to evacuate the liquid, it becomes detached and flows in greater abundance than you would have believed, and, the walls of the neck collapsing, it appears to be emptied. When you apply the speculum—and this is useful—you must use that with two valves, by means of which, after having placed it properly and seized the neck between the two gutters formed by the valves of the instrument, you may make repeated pressure, and cause to flow abundantly, and almost in a jet, the liquid contained in the cavity of the organ and in the sacs of the mucous follicles. The quantity of the mucus contained in the neck may be considerable, and may give rise to what M. Jobert calls dropsy of the neck, which in our opinion is but a form of catarrh. In effect, the liquid is simply accumulated in the cavity of the neck, and not contained in a closed and distinct sac.

In certain cases, the mucus secreted by the follicles of the cavity of the uterus is in communication with the liquid of the same nature furnished by the follicles situated on the surface of this organ which is in contact with the vaginal walls, and gives rise to more or less considerable masses of fluid. Whatever may be the point of the vagina where this deposit takes place, it is easy to see that the matter which constitutes it is not the product of the mucous membrane of the vagina, but rather of the glandular apparatus of which we have spoken. In fact, we always find a stream of liquid which is directed towards the neck, the place of its origin; and it is thus that the mass of the liquid has glided to the vulvar opening. It sometimes happens that, in consequence simply of the vertical position, the mass of liquid becomes suddenly detached and falls to the ground. It is not in this way that the discharges in disorders of a different character are effected; in these, the escape of the fluid is never in a body or mass, but slowly, and drop by drop.

Let us now say something on the appearance presented by the mucus of uterine catarrh deposited upon the linen. The stains which this liquid forms upon the linen are quite round, and not angular as is the case in those formed by discharges from the vagina; they glue the linen, rendering it firm as if starched; their color is a dull white, grayish, or greenish yellow. It is important to be able clearly to distinguish these taches or spots. In the first place, it may be of some importance in a medico-legal investigation; then it may happen that you will meet with certain women who will not allow any examination whatever of their genital organs, and you are thus obliged to put up with an examination of their linen; and these, too, are difficulties which you must expect to meet but too often in practice.

2. *Chemical Characters.*—If we look for a moment at the chemical properties of the liquid of which we are speaking, we shall see that it offers characters peculiar to itself. This liquid, in fact, is alkaline; it may sometimes show itself acid, but this is then owing to its mixture with the products of a secretion of a different nature and source; so that if it were possible to separate and analyze apart the liquids mixed together, that dependent upon catarrh of the uterus would always present an alkaline character. When you collect and set aside this liquid to dry, it becomes solid, brittle, elastic; put it in water, where it does not dissolve, it regains all the characters by which it was recognised in the liquid state. The matter of discharges from the genital organs, other than that belonging to catarrh, does not present the same chemical and physical characters as the latter. Thus we find among them some differential characters which are of no mean value.

3. *Contagious Properties.*—We proceed to add to the foregoing some brief considerations upon the contagious properties of the liquid produced by uterine catarrh, which is a question not altogether devoid of interest. Catarrh of the uterus is evidently contagious when the consequence of or when accompanied by, a syphilitic disease; it may be contagious when it is simply blennorrhagic, without being syphilitic. When blennorrhagic catarrh has continued a certain length of time, it may lose its contagious properties, as you are aware, in urethral blennorrhagia.

A urethritis in a man may be the consequence of sexual connection with a woman laboring under a simple non-blennorrhagic catarrh. It is not very rare to see in the same woman a catarrh showing itself contagious at certain times, and not constantly. This may be owing to certain transient modifications produced in the matter of the discharges in certain women placed accidentally in diverse conditions, and inappreciable circumstances. It is thus that excess in coition, menstruation, fatigue, and other causes, may develop a principle of acidity in the product of the catarrhal secretion, and give to it contagious properties; these transient causes subsiding, the effects subside also, and the woman becomes exempt from all contagious qualities. Nor is it unexampled for women, laboring under uterine catarrh, to fail to communicate disease to the men with whom they have habitual intercourse, and yet produce a blennorrhagia in those who have had with them but occasional connections. It is important to be aware of facts such as these; their explanation is perhaps difficult, but their existence is not the less incontrovertible, and this should suffice us.

*March and Duration.*—Catarrh of the uterus maintains a continued march; no intermissions mark its progress. Nevertheless menstruation exercises an appreciable influence upon this affection. In effect, two or three days before the menses appear, the catarrh is often seen to diminish, then to be suspended, and suppressed during their continuance, to reappear after their cessation, and resume its continued march; it acquires sometimes a greater intensity after the menses, though this is but a momentary result.

Catarrh of the uterus may present itself under two forms—acute and chronic. In the first case, it may terminate at the end of a month or a month and a half. But the chronic form is the most frequent, and the disease then may last for many months or years, and even during the whole of the period of life in which the woman is susceptible of fecundation, it rarely continuing after the final cessation of the menstrual discharge.

*Symptoms.*—When the catarrh is chronic, essential, and not complicated, it gives rise only to unimportant functional troubles. There is remarked a vague general uneasiness, some sensations of twitching in the groin, in the vagina, in the interior of the womb; sometimes indigestion; a little loathing, sadness, etc.; and some complications may also occur. Thus there exists sometimes an inflammatory condition of the parts, of the neck itself, the surface of which may be red, bleeding, and deprived of epithelium, and flabby and fungous around the follicles. The orifice of these follicles may be enlarged and ulcerated. The reactional troubles will be more or less marked according to the complications, they being in fact in a ratio with the nature and degree of intensity of the phenomena of complication.

*Diagnosis.*—We have seen what are the characters proper to discharges of catarrh of the uterus; we find these the essential element of the diagnosis of the affection. But you must not neglect to inquire if the disease is simple; if it is owing to a local or general cause; if it is connected or not with an affection of the tissue of the neck or of the body of the uterus; if it is the consequence of a lymphatic temperament, of an anemic state, of a scrofulous taint, etc.—circumstances all calculated to furnish essential therapeutic indications.

*Acute Pleurisy. Thoracentesis.*—Although acute pleurisy is not ordinarily an extremely grave disease, there are cases where the termination is fatal. Respecting these terminations M. Trousseau has the following interesting remarks:

1. Acute pleurisy may kill by the excess of effused liquid. 2. It may kill as a phlegmasia, independently of the quantity of the effusion. 3. In the same individual there may be an excess of effusion capable of killing independently of the reactional influence of the phlegmasia, and a phlegmasia capable of producing death independently of the excess of effusion.

Struck with the manner in which acute pleurisy may become fatal in the first category, M. Trousseau demanded if, in these circumstances, it would not be possible to evacuate the liquid by means of a surgical operation, as is sometimes done in chronic pleurisy. In 1843 he acted upon this thought, and had the good fortune to succeed. In 1844 he twice repeated the operation; in 1845 he again performed it; and in a memoir published in the *Journal de Médecine*, he has based upon the three propositions above given the three following therapeutic conclusions: 1. In the first case, paracentesis of the thorax is a powerful means, exempt from danger. 2. In the second case, thoracentesis is at least hurtful. 3. In the third, it is useful, as it certainly prolongs life, and thus opens up some chance to the patient.

M. Trousseau, we see in this, does not recommend thoracentesis as a common operation, to be performed daily, like venesection, cupping and blistering, but as an exceptional method which is only applicable in certain rare cases. This operation of thoracentesis in a case of acute pleurisy was performed by M. Cullerier a few weeks ago in one of the wards at the Hotel Dieu, in which I was taking one of my private courses. A recapitulation of the case may be interesting:

A short time since a man forty-three years of age entered the hospital, laboring under a simple acute pleurisy. A bleeding at the arm and two blisters effected a notable amelioration of the symptoms and considerable diminution of the effusion. Suddenly, without any known cause—probably in consequence of some imprudence of the patient—the inflammation appeared with increased intensity; the chest filled anew; stitch in the side, and intense febrile action. Cups were applied, followed by four successive vesicatories, and purgatives. Notwithstanding these energetic means, the effusion, far from diminishing, seemed to augment; the dyspnœa was greater; the fever alone had abated. The patient, although he had here evidently been attacked with an acute inflammation of the pleura, was, as far as his general condition was concerned, in the same state as if he had been attacked with chronic pleurisy. He was in the following condition when the operation was performed: The right side was enormously dilated; in the inferior half of the chest there was œdema of the soft parts of the walls of the thorax; dullness throughout the whole of the right side. The liver was manifestly pushed back into the iliac fossa, and percussion gave an obscure sound until about two inches above the crural arch. Slight œdema of the right thigh; extreme dyspnœa; respiration very rapid; scarcely any febrile movement.

M. Cullerier commenced the operation by making with a bistoury an oblique incision of the skin, which comprehended also the muscles, from above downwards and from behind forwards, in the direction of the intercostal space, between the eighth and ninth ribs. Arrived upon the pleura, the surgeon penetrated into the thoracic cavity with a trocar, introduced at the inferior angle of the wound. The first quart of liquid evacuated consisted of a yellowish serum, a little turbid, resembling in color urine slightly changed. The patient having now changed his position, the liquid which flowed by the canula changed its nature, and of ten pounds, which was about the amount of the effused fluid, three-quarters at least were composed of almost pure pus. The operation was barely completed when the liver returned to its normal position. The day of the operation and during the whole of the next, pus escaped by the opening made in the walls of the thorax. M. Cullerier carefully introduced, on the second day, a sound into the wound; this maneuver gave issue to more than a quart of sero-purulent liquid. A blister was applied upon the diseased side, with the view of creating an obstacle to the production of any new serum. It is now eight days since the operation, and, save a slight access of fever that arose on the fourth day, everything has gone on well.

It is evident that thoracentesis must remain an exceptional method, and that its indications are in nowise so common as those of the other means that are daily employed against acute pleurisy. M. Trousseau has expressed, in a few lines, the indications which warrant it: "When the dullness shall be perceived in front and above, and shall have attained the median line from the sternal curve to the fourth rib, and the diaphragm shall have attained its greatest degree of depression, it will be desirable to make the puncture, although there may be no urgent necessity. But if, notwithstanding the activity of the means employed, the dullness shall pass beyond the median line, and continues each day to extend towards the opposite side to the extent of four or five millimetres, there is urgent necessity for puncture; and it is still more pressing if the effusion is on the left side. And when the effusion has not passed the median line, if the orthopnœa is extreme, the pulse small and very frequent, the face excessively anxious, especially if there is a tendency to lypothymia, it is also necessary to operate as soon as possible."

An opinion generally accredited among physicians is, that the pneumonia which supervenes during the course of pulmonary phthisis is always or almost always mortal. What has probably contributed to extend this opinion is, that in a great number of persons who have died of phthisis, there are found at the autopsy some equivocal traces of acute inflammation of the pulmonary parenchyma. But a distinction should be established between the pneumonia which supervenes in the last period of pulmonary phthisis, and that which is observed often during the first periods of the evolution of tubercles. And in this last case, it is still necessary to distinguish between the tuberculous pneumonia of the child and that of the adult. In the child it is always mortal; already very grave in itself, the pneumonia seems to be a spur which imparts a more rapid march to tuberculization. In a very little time, the symptoms become intensely acute, and death occurs almost immediately. In the adult, it is far from being the same. The phlegmasia of the pulmonary parenchyma is quite common at an early period of the tuberculous disease; and in this case it is remarkable that it possesses but little gravity, and that it most frequently terminates in a favorable manner. Extraordinary as it may appear, the fact is not the less certain, the observations of Louis, Andral and Grisolle having put it beyond a doubt. The last named gentleman, who has published the most complete treatise on pneumonia extant, has proved, moreover, that the pneumonia which supervenes at an early period of phthisis does not appear to aggravate, at least as a general rule, the first disease—a proposition which M. Louis, since the publication of his work on phthisis, has recognised as correct. Most physicians have had more than one opportunity of verifying the almost perfect harmlessness, or at least comparatively slight gravity, of pneumonia supervening among subjects manifestly attacked with tubercles in a slightly advanced state. A few days since there was a case of this kind in the wards of M. Louis at the Hotel Dieu. The explanation which he gives of this fact, at first apparently so strange, is, that the pneumonia which supervenes in these circumstances is in some degree analogous to traumatic pneumonia, of comparatively mild nature, as we know; and that tubercles in this case simply act the part of a foreign body.

Paris, July, 1847.

